# Cell Type-Specific Expression of Purinergic P2X Receptors in the Hypothalamus

**DOI:** 10.3390/ijms26115007

**Published:** 2025-05-22

**Authors:** Jana Cihakova, Milorad Ivetic, Hana Zemkova

**Affiliations:** 1 Institute of Physiology, Czech Academy of Sciences, CZ-142 20 Prague, Czech Republic; jana.cihakova@fgu.cas.cz (J.C.); milorad813@gmail.com (M.I.); 2 First Faculty of Medicine, Charles University, CZ-121 08 Prague, Czech Republic

**Keywords:** supraoptic nucleus (SON), paraventricular nucleus (PVN), arcuate nucleus (ARC), suprachiasmatic nucleus (SCN), hypothalamus, P2X, extracellular ATP

## Abstract

Purinergic P2X receptors (P2X) are ATP-gated ion channels that are broadly expressed in the brain, particularly in the hypothalamus. As ionic channels with high permeability to calcium, P2X play an important and active role in neural functions. The hypothalamus contains a number of small nuclei with many molecularly defined types of peptidergic neurons that affect a wide range of physiological functions, including water balance, blood pressure, metabolism, food intake, circadian rhythm, childbirth and breastfeeding, growth, stress, body temperature, and multiple behaviors. P2X are expressed in hypothalamic neurons, astrocytes, tanycytes, and microvessels. This review focuses on cell-type specific expression of P2X in the most important hypothalamic nuclei, such as the supraoptic nucleus (SON), paraventricular nucleus (PVN), suprachiasmatic nucleus (SCN), anteroventral periventricular nucleus (AVPV), anterior hypothalamic nucleus (AHN), arcuate nucleus (ARC), ventromedial hypothalamic nucleus (VMH), dorsomedial hypothalamic nucleus (DMH), tuberomammillary nucleus (TMN), and lateral hypothalamic area (LHA).> The review also notes the possible role of P2X and extracellular ATP in specific hypothalamic functions. The literature summarized here shows that purinergic signaling is involved in the control of the hypothalamic-pituitary endocrine system, the hypothalamic–neurohypophysial system, the circadian systems and nonendocrine hypothalamic functions.

## 1. Introduction

**Figure 1:**
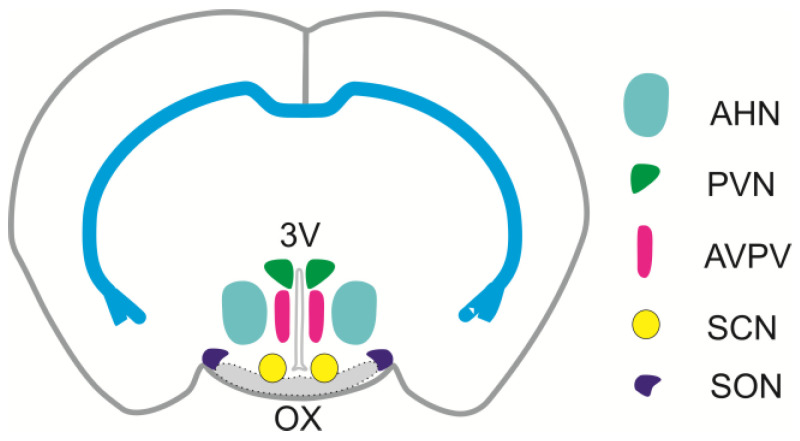
Structure of anterior hypothalamus showing anterior hypothalamic nucleus (AHN), paraventricular nucleus (PVN), anteroventral periventricular nucleus (AVPV), suprachiasmatic nucleus (SCN), supraoptic nucleus (SON), optic chiasm (OX), and the third ventricle (3V).

The hypothalamus is the part of the brain that is functionally connected to the limbic system and pituitary gland. This relatively small area of the brain contains a number of small nuclei, which play a key role in coordinating fundamental body functions. Particularly, the hypothalamus has important functions in maintaining the homeostasis of body fluids, regulating the blood flow, lactation, parturition, body temperature, appetite, systemic metabolism, growth, sleep and waking cycle, and social behavior [1,2]. Anatomically, the hypothalamus can be arranged into three main regions: the anterior (Figure 1), middle, and posterior hypothalamus. Each major region contains nuclei that serve certain physiological functions. The anterior region contains at least five important nuclei: supraoptic (SON), paraventricular (PVN), suprachiasmatic (SCN), anteroventral periventricular (AVPV), and anterior hypothalamic nucleus (AHN). The middle region contains three nuclei: the arcuate nucleus (ARC), the ventromedial hypothalamic (VMH), and dorsomedial hypothalamic (DMH) nucleus. The posterior region contains the tuberomammillary nucleus (TMN) and the lateral hypothalamic region (LHA), which serves for the integration of autonomic and limbic information. Hypothalamic nuclei contain many molecularly defined neuronal populations that synthesize neuropeptides alongside classical neurotransmitters such as γ-aminobutyric acid (GABA) and glutamate. Molecular characterization of neuronal subtypes is presented in an integrated reference atlas, “HypoMap”, which provides a comprehensive single-cell transcriptomic atlas of the mouse hypothalamus and reveals transcriptomic diversity of cell types in different hypothalamic nuclei [3].

The hypothalamus is known to abundantly express purinergic P2X receptors (P2X) activated by extracellular adenosine-5′-triphosphate (ATP). Hypothalamic neurons express several subtypes of P2X [4], which are also expressed in hypothalamic astrocytes [5,6], tanycytes [7,8] and microvessels [9]. Although purinergic signaling has a well-established role in the regulation of energy homeostasis and feeding behavior [10], and extracellular ATP is known to be involved in regulating the sleep–wake cycle [11], the role of P2X in hypothalamic functions is not yet fully understood. Several reviews described the expression and function of P2X and purinergic signaling pathways in the hypothalamic–neurohypophysial system [5,12], the hypothalamic–adenohypophysial system [13,14,15], endocrine glands [16,17], and circadian system [18,19,20]. This review focuses on the distribution of P2X in the most important hypothalamic nuclei, and the possible involvement of extracellular ATP and P2X in endocrine and non-endocrine hypothalamic functions.

## 2. Nuclei and Neuron Types in the Hypothalamus

### 2.1. Supraoptic Nucleus (SON)

The supraoptic nucleus (SON) is located at the bottom of the anterior hypothalamus (Figure 1), adjacent to the chiasma opticum and in the vicinity of hypothalamic blood vessels. Its shape and size depend on the species; it can be oval shaped or elongated, and in humans, it stretches through most of the ventral side of the hypothalamus [21]. SON contains two types of magnocellular neurons that synthesize and secrete two hormones: oxytocin and vasopressin, also called arginin-vasopressin (AVP). The axons of magnocellular neurons transport oxytocin and vasopressin stored in large dense-cored vesicles from the soma to the posterior pituitary, where both hormones are secreted into the systemic circulation. The main function of oxytocin and vasopressin is to regulate childbirth and breastfeeding, and to maintain the homeostasis of body fluid, respectively. Oxytocin stimulates the contraction of myoepithelial cells in the lactating mammary gland and triggers uterine contractions during labor; the release of oxytocin is mainly caused by parturition and suction reflex [22,23,24,25]. Vasopressin serves to regulate plasma osmolality and blood pressure; it acts on the kidney to increase water reabsorption and causes vasoconstriction. The release of vasopressin increases as a function of plasma osmolality and water status of the body [26,27,28]. Furthermore, oxytocin and vasopressin are also released, via dendritic release [29,30], into the blood vessels inside the brain [21]. Through the actions at the level of the CNS, via their specific receptors on neurons, oxytocin and vasopressin are independently involved in facilitating of maternal and parental care, and other social behaviors [31,32,33,34]. The region that expresses the oxytocin receptor and controls maternal behavior, for example, is the medial preoptic area (MPOA). Oxytocin and vasopressin are also released within the SON itself, where they have local regulatory effects [35,36,37].

The secretion of oxytocin and vasopressin is dependent on the electrical activity of magnocellular neurons [38], which has an in vivo specific pattern for oxytocin and AVP neurons [22,23,25,27,28,39]. Both hormones are released in response to the activity-dependent Ca^2+^ influx, which primes large dense-cored vesicles for exocytosis [28,40,41,42,43]. Magnocellular neuron activity is controlled by various excitatory and inhibitory synaptic inputs [27,44,45,46]. These inputs use glutamate [47,48,49,50] and γ-aminobutyric acid (GABA) [24] as the main neurotransmitter. Other inputs use norepinephrine, serotonin, dopamine, histamine, acetylcholine, angiotensin II, nitric oxide, β endorphin, and ATP (for a review, see [51]).

### 2.2. Paraventricular Nucleus (PVN)

The paraventricular nucleus (PVN) is located on the sides of the third ventricle of the hypothalamus, at the top of the ventricle (Figure 1). It is one of the most important neuroendocrine and autonomic regulatory brain centers, with neurons playing an essential role in controlling stress, metabolism, growth, reproduction, immune, gastrointestinal, renal, and cardiovascular functions. The PVN receives afferent inputs from many regions which are then reflected in changes in single specific outputs [52]. By function, the PVN contains three types of peptidergic neurons, predominantly neuroendocrine: magnocellular, parvocellular, and preautonomic (for a review, see [53]). Anatomically, the PVN of the rat consists of three parts: the dorsal and medial parts contain mainly parvocellular neurons, and the posterior part contains magnocellular neurons [54,55]. Firstly, magnocellular neurons have large cell bodies due to their necessary large capacity for peptide synthesis and secretion, while parvocellular neurons are small, reflecting less need for production of less stored peptide [1]. Further, magnocellular PVN neurons project to the posterior pituitary, where they release oxytocin and vasopressin directly into the general circulation. These neurons receive similar afferent inputs as magnocellular oxytocin and vasopressin neurons in the SON [51]. Secondly, parvocellular neurosecretory cells synthesize hypophysiotropic hormones and project to the median eminence. These hormones are secreted into the hypothalamic–hypophysial portal system, and transported vascularly to the anterior pituitary gland, where they cause the immediate release of pituitary hormones. Parvocellular neurons produce and secrete corticotropin-releasing hormone (CRH), which regulates secretion of adrenocorticotropic hormone (ACTH); thyrotropin-releasing hormone (TRH), which controls secretion of thyroid-stimulating hormone (TSH); and somatostatin and growth hormone-releasing hormone (GHRH), which both control secretion of growth hormone (GH). The majority of these neurons lie in the medial parts of PVN. In addition, parvocellular preautonomic neurons project to autonomic centers in the brain stem and spinal cord [54,56,57].

Each of these PVN neuron types, especially the CRH, oxytocin, vasopressin, somatostatin, and TRH neurons, are also centrally projecting neurons releasing peptides in hippocampus, locus coeruleus, lateral septum, and nucleus accumbens, for example [1], and affect many types of behavior in mice and humans [31,32]. For example, CRH plays a critical role in the brain’s response to stress, emotional regulation, and other physiological processes associated with sleep, learning, memory, cognition, food intake, and motor co-ordination. Dysregulation of the CRH system has been associated with neurological and psychiatric disorders, including anxiety and depression [53,58]. Next, oxytocin neurons in the PVN are involved in the regulation of empathic [59,60] and feeding behaviors [61]. Retrograde tracer analysis has shown that projection of vasopressin neurons from SCN to PVN links light perception to feeding behavior, so that light exposure acutely suppresses food intake and increases c-Fos expression in the oxytocin neurons of PVN; the light-induced suppression of food intake was mostly abolished by blockade of the oxytocin receptor [61]. Last, TRH plays a role in the central control of food intake so that the central administration of TRH may produce anorexigenic or orexigenic effects, depending on the injection site of TRH [62].

A group of preautonomous parvocellular neurons that do not belong to the endocrine neurons and are involved in the central control of the autonomic nervous system include neurons releasing angiotensin II (AngII) and nitric oxide (for an overview, see [63]).

### 2.3. Suprachiasmatic Nucleus (SCN)

The suprachiasmatic nucleus (SCN) is located directly above the crossing of optic nerves, on both sides of the third ventricle (Figure 1). It represents a heterogeneous structure of ~10,000 GABA-producing neurons accompanied by a large number of glial cells [64]. The SCN is a principal internal time-keeping system that synchronizes the behavior and physiology of an entire organism with a 24 h cycle [65,66,67], and helps to establish the normal sleep–wake cycle by influencing the synthesis and release of melatonin from the pineal gland. Endogenous circadian rhythms are driven by the expression of clock genes in each SCN neuron [68]. It regulates the circadian rhythms of various physiological processes, including motor activity, body temperature, plasma hormone levels, renal secretion, and metabolism.

The SCN contains two types of neurons and, anatomically, it can be divided into dorsomedial (shell) and ventrolateral (core) parts [69]. The light-responsive neurons producing vasoactive intestinal peptide (VIP) are found in ventrolateral part of the SCN, while the endogenously rhythmic neurons producing AVP are found in the dorsomedial region of this nucleus, which does not receive a direct input from the retina [70,71,72]. Intercellular communication between endogenously rhythmic neurons and light-responsive neurons is likely responsible for the unique pacemaker properties of the SCN observed at a tissue and whole-body level [69]. Finally, the circadian rhythm of AVP secretion runs parallel to the rhythm of electrical activity in SCN neurons [73,74,75]. Using AVP secretin, the SCN interacts with other regions of the brain to synchronize the circadian rhythms of various physiological processes. For example, retrograde tracer analysis has shown that the projection of AVP neurons from the SCN to PVN links light reception to feeding behavior [61].

### 2.4. Anteroventral Periventricular Nucleus (AVPV)

The anteroventral periventricular nucleus (AVPV) belongs to the preoptic region (POA), a broader area encompassing several nuclei, including the periventricular nucleus (PeN), median preoptic nucleus (MnPO), and medial preoptic area (MPOA). The AVPV, specifically located in the hypothalamus (Figure 1), is a small cluster of cells that is characterized by its sexually dimorphic structure, being larger in females than in males. The AVPV is particularly important as the region containing neurons producing gonadotropin-releasing hormone (GnRH, also called luteinizing hormone-releasing hormone, LHRH) and neurons producing kisspeptin, which are both involved in the regulation of fertility and production of gonadal steroid hormones [76].

Firstly, GnRH neurons regulate reproductive function in mammals by inducing synthesis and release of follicle stimulating hormone (FSH) and luteinizing hormone (LH) from the anterior pituitary gland. They are extremely rare, elongated, bipolar or unipolar and, in rodents, the cell bodies of GnRH neurons are diffusely distributed throughout the basal forebrain. The loosely organized population of GnRH cells receives neuronal inputs from many (>50) different brain regions [77]. One or two very long dendritic processes of GnRH neurons are located primarily in the region around the organum vasculosum of the lamina terminalis (OVLT), and their nerve terminals are mainly found in the median eminence [78]. In the AVPV, GnRH is released in pulses at hourly intervals, and this pulsatility is essential for the maintenance of the reproductive function of adult animals [79]. In addition, GnRH is also released in various regions of the CNS, and affects neurological disorders, neuroprotection, cognitive functions, and mood regulation [80].

Secondly, kisspeptin neurons are located near GnRH neurons and their dendrons, and produce neuropeptide kisspeptin, which activates the hypothalamic–pituitary–gonadal axis and influences neural circuits controlling sexual behavior [81]. Kisspeptin acts through specific kisspeptin receptors (Kiss1R, also known as GPR54) expressed by GnRH neurons [82] to induce the synthesis and release of GnRH and, consequently, LH surge and ovulation [83]. Furthermore, kisspeptin neurons express estrogen receptors that play a well-established role in the estrogen-dependent regulation of reproduction [76]. AVPV kisspeptin neurons are primarily involved in the estrogen-induced positive feedback mechanism, which triggers the preovulatory luteinizing hormone (LH) surge in females [76]. For example, *Kiss1* (kisspeptin gene)- and *Gpr54* (kisspeptin receptor gene)-deficient mice, rats, and humans are infertile and suffering for hypogonadotropic hypogonadism [83]. AVPV kisspeptin neurons project to neuronal nitric oxide synthase (nNOS) producing neurons in the ventrolateral part of the ventromedial hypothalamus (VMH) to stimulate lordosis behavior. Linking of kisspeptins to metabolism and reproduction has been recently reviewed at [84]. In addition, kisspeptin is also produced by kisspeptin neurons in the ARC.

### 2.5. Anterior Hypothalamic Nucleus (AHN)

The anterior hypothalamus (AHN) is a specific nucleus that controls body temperature. About 30% of AHN neurons are warm-sensitive, many of them receiving inputs from afferent pathways that communicate information from skin thermoreceptors [85].

### 2.6. Arcuate Nucleus (ARC)

The arcuate nucleus (ARC), located in the middle hypothalamic region, contains diverse populations of neurons that participate in the control of food intake, body weight [86,87], energy homeostasis [88], feeding behavior, blood pressure, and cardiovascular function, particularly in the context of obesity and metabolic disorders (for a review, see [89]). Neurons in the arcuate nucleus synthesize the neuropeptide Y (NPY), agouti-related peptide (AgRP), proopiomelanocortin (POMC), cocaine and amphetamine-regulated transcript (CART), and the α-melanocyte-stimulating hormone (α-MSH), according to the nutritional status of the organism. Their nerve endings in the PVN activate or inhibit neurons synthesizing TRH to control food intake [1]. ARC neurons also control the endocrine functions of the anterior pituitary gland by producing both releasing and inhibiting neurohormones secreted in the median eminence. These include growth hormone-releasing hormone (GHRH) and somatostatin, which regulate the hypothalamic–pituitary–growth hormone axis, and prolactin-inhibiting hormone (dopamine), which regulates the hypothalamic–pituitary–prolactin axis.

Arcuate nucleus contains GnRH neurons producing GnRH, which regulates hypothalamic–pituitary–gonadal axes in a sex-specific manner [1] and, in rodents, neurons that produce kisspeptin [90]. ARC kisspeptin neurons mediate negative feedback regulation of GnRH and LH secretion by estrogen, and they are crucial for generating pulsatile GnRH/LH release in female mammals [91]. Simultaneously, kisspeptin neurons coexpress neurokinin B (NKB) and dynorphin A (DYN); therefore, they are termed KNDy neurons that regulate rhythmic GnRH pulses essential for reproductive cyclicity. The pulsatility of GnRH release is markedly reduced in aged animals, suggesting that the malfunctions of ARC kisspeptin neurons may be responsible for reproductive aging and menopause-related disorders [92,93].

In addition, ARC neurons have contact with neurons in PVN [94] and AVPV [95], where they regulate the production of the CRH and GnRH, for example.

### 2.7. Ventromedial Hypothalamic Nucleus (VMH)

The ventromedial nucleus of the hypothalamus (VMH) is also located in the middle hypothalamic region. It has been implicated in the regulation of a wide range of physiological and behavioral processes, including feeding, sexual and maternity behavior, blood calcium homeostasis, circadian neuroendocrine control, hyper-running physical activity, and emotional behavior [1]. The VMH was considered the brain’s center of satiety or fullness [96]. For example, lesions of the VMH result in hyperphagia and obesity in a variety of species including humans (for a review, see [97]). The majority of VMH neurons express steroidogenic factor 1 (SF-1), an orphan nuclear receptor, which was identified as a key regional marker of VMH neurons in the HypoMap Atlas [3]. Neurons producing SF-1 play a critical role in regulating energy homeostasis, glucose metabolism, and body weight [98,99,100,101], which highlights SF-1 neurons as potential targets for anti-obesity therapies.

### 2.8. Dorsomedial Hypothalamic Nucleus (DMH)

The dorsomedial nucleus of the hypothalamus (DMH) belongs to complex neuronal network that integrates incoming nutrient and hormonal signals and gates neuronal output to maintain homeostasis. Among feeding circuits, the DMH is recognized as a key player in the regulation of appetite [102]. Further, the DMH is also involved in control of circadian rhythms, thermoregulation (including the control of brown adipose tissue thermogenesis) and autonomic functions [1], regulation of neuroendocrine stress responses [103], and the cardiovascular response associated with emotional stress [104]. Stimulation of this nucleus, in animal experiments, produced aggressive behavior that lasts only as long as the stimulus is applied. The DMH receives inputs predominantly from adjacent hypothalamic nuclei, notably the VMH and POA, and in turn projects into PVN, for example [102].

### 2.9. Tuberomammilary Nucleus (TMN)

The tuberomammillary nucleus (TMN), located in the posterior hypothalamus, consists primarily of histaminergic neurons, which are involved in memory, changes in emotion, and heart failure. Activity of histaminergic TMN neurons is closely associated with the sleep–wake cycle. These neurons are tonically activated during wakefulness, just a little activated during slow wave sleep, and not at all activated during rapid-eye-movement (REM) sleep [105]. In addition, the TMN receives inputs from the pre-frontal cortex and the POA, while its efferents reach most parts of the brain [106].

### 2.10. Lateral Hypothalamic Area (LHA)

The lateral hypothalamic area (LHA), also called the lateral hypothalamus (LH), is primarily located in the lateral hypothalamic region, and is considered part of the posterior hypothalamus. It is heterogeneous region, containing many molecularly defined neuronal populations that mediate distinct aspects of physiology, including energy balance and feeding, arousal and sleep, stress and reward, body temperature, and autonomic functions [107]. The LHA contains three main types of neurons. Firstly, the dynorphin peptide (DYN) neurons [108], which are involved in the food intake, appetitive motivation and modulation of feeding-related behaviors such as arousal. DYN is also the endogenous ligand for the kappa opioid receptor (KOR), which regulates food intake [109]. Secondly, hypocretin/orexin neurons, which project extensively throughout the CNS, influencing multiple brain regions involved in wakefulness [110,111]. LHA-derived orexinergic neurons also project into neuroendocrine centers, which are known to be important for the physiological response to stress [112]. Dysfunction of the orexin/hypocretin neurotransmitter system leads to the sleep disorder narcolepsy. Thirdly, the LHA is responsive to noxious stimuli [113], and a large population of neurotensin-expressing neurons in the LHA project to brain regions that participate in descending control of pain processing [114].

## 3. Sources of Extracellular ATP in the Brain

**Figure 2:**
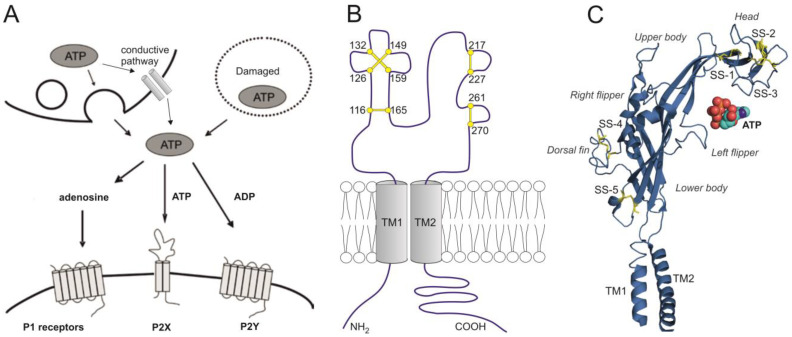
Extracellular ATP and molecular structure of P2X receptors. (**A**) ATP is released both from damaged and healthy cells. From healthy cells, ATP is released through exocytosis and conductive pathways, such as connexin and pannexine semichannels, volume-regulating channel, large anion channel, and purinergic P2X7 channel, or transporters such as VNUT. (**B**) Each of the P2X subunits has two transmembrane structures (TM1 and TM2) with both N- and C-terminus located intracellularly. All P2X receptors contain 10 conserved cysteine residues in their large extracellular domains. Five disulfide bonds are formed between the following cysteine residues (rat P2X4 numbering):116–165 (SS-1), 126–149 (SS-2), 132–159 (SS-3), 217–227 (SS-4), and 261–270 (SS-5). (**C**) Secondary structure organization of one zebrafish P2X4.1 receptor subunit [145] with α-helices, β-sheets and turns (in blue), five disulfide bonds (in yellow), and one ATP molecule (PyMol; pdb 3I5D). The ATP binding pocket lies between the P2X subunits, so the trimeric receptor has three ATP binding pockets. The structure of the P2X subunit has been compared to that of a dolphin: following ATP binding, the head, upper body, and left flipper domains of one subunit, and the lower body and dorsal fin domains of another subunit undergo significant movement resulting in the closure of the ATP binding site jaw [146].

In the brain, ATP is released during normal neuronal activity, but high extracellular ATP levels occur at pathological brain states, such as seizures [115], hypoxia [116], or Alzheimer’s disease [117]. Large amounts of ATP is also released from damaged or dead cells. The main source of extracellular ATP in the brain are astrocytes [118,119,120,121,122], which play an important and active role in neural function as a participant in the “tripartite synapse” [123]. High frequency stimulation-induced elevations in extracellular glutamate could trigger a sufficient increase in intracellular astrocytic Ca^2+^ to stimulate ATP release [124]. ATP was detected in large amounts in dense-core vesicles and in lysosomes of astrocytes [125]. Consequently, ATP is released from astrocytes into the synaptic cleft, where it can act on presynaptic or postsynaptic P2 receptors [126,127,128] to modulate the release of neurotransmitters [129,130,131,132,133,134] or postsynaptic efficacy [135]. ATP is released from astrocytes by the mechanism of Ca^2+^-regulated exocytosis [136,137,138], or through conductive pathways (Figure 2A), such as pannexin-1 hemichannels [139,140,141,142] or P2X7 channels [143]. ATP may also be released via astrocyte-selective vesicular nucleotide transporter (VNUT) [144].

## 4. A Brief Overview of Purinergic P2X1-7 Receptors

Purinergic P2X receptors (P2X1-7) are ligand-gated ion channels activated by extracellular ATP. Each P2X subunit has two transmembrane domains, a long cysteine-riched extracellular ligand-binding domain, and the N-terminal and C-terminal regions located intracellularly (Figure 2B,C) [147]. Three P2X subunits form homomeric and/or heteromeric ion channels [148]. P2X sometimes even interact with other ion channels; for example, P2X7 interacts with pannexin-1 hemichannel [149], P2X4 with GABA receptor type A (GABA_A_ receptor) [150,151,152], and P2X5 with acid-sensing ion channel 3 (ASIC3) signaling pathway [153]. P2X receptors present almost equal permeability to N^+^ and K^+^, and substantial calcium permeability [154,155,156]. When expressed in *Xenopus* oocytes or human embryonic kidney cells (HEK), the homomeric P2X1-7 subtypes differ in sensitivity to agonists, antagonists and allosteric modulators, ion selectivity, and desensitization rate [147,157,158] (see Table 1).

Most P2X exhibit sensitivity to the inhibitory effects of suramin and pyridoxalphosphate-6-azophenyl-2′, 4′-disulfonic acid (PPADS) [147]. However, many new selective antagonists are currently available for different P2X subtypes (for a review, see [159,160,161]). Under physiological conditions, P2X can be modulated by a wide array of native molecules and allosteric modulators such as protons, trace metals like zinc and copper, phosphoinositides, alcohols, reactive oxygen species [147,159,161], and neurosteroids [162].

**Table 1:** The major functional and pharmacological properties of P2X1-P2X7 receptors.

Receptor	Desensitization Rate	Ca^2+^ Permeability	αβme- ATP	Suramin PPADS	IVM	Acid pH	Large Pore
P2X1	Fast	High	Yes	Yes	No	Inhibited	No
P2X2	Slow	Medium	No	Yes	No	Potentiated	Yes
P2X3	Fast	Low	Yes	Yes	No	Inhibited	No
P2X4	Medium	High	No	Low	Yes	Inhibited	Yes
P2X5	Slow	Low	No	Yes	No	No	No
P2X6	N/D	N/D	N/D	N/D	N/D	N/D	N/D
P2X7	None	High	No	Low	No *	Inhibited	Yes

* Ivermectin (IVM) acts as a positive allosteric modulator not only for P2X4, but also for human P2X7 [163]. N/D, not determined.

Here is a brief overview of functional characteristics and pharmacology of P2X1-7 subtypes.

**P2X1** has the highest affinity for ATP, desensitizes more quickly compared to other P2X subtypes, in the millisecond range, and can be activated by synthetic ligands such as α,β-methyleneadenosine 5′-triphosphate (αβmeATP) [147,164]. In addition, homomeric P2X1 belongs to P2X that exhibit the highest relative Ca^2+^ permeability among P2X [156]. P2X1 is expressed namely in urinary bladder and vas deferens, where they are known to have a role in contraction of smooth muscles, or in neutrophil chemotaxis or thromboinflammation [147,165].

**P2X2** exhibits moderate sensitivity to ATP, and is considered to be a non-desensitizing receptor channel selectively permeable to cations and large organic cations, such as N-methyl D-glucamine (NMDG^+^) [166,167]. P2X2 can be potentiated by acidic pH and zinc [168] and inhibited by PPADS and suramin [169], but up to date, no P2X2-specific inhibitor has been found. Studies on the *P2rx2r* reporter mouse models have shown that P2X2 is widely distributed in the CNS [170,171,172], where it is present in different splice variants including the P2X2b, P2X2c, P2X2d, P2X2e, P2X2f, and P2X2g [173]. The presynaptic P2X2 have been shown to underlie an increase in GABA release in a subset of GABAergic interneurons in the spinal cord [174]. Further, experiments with knockout mice have shown that Ca^2+^ entry through presynaptic P2X2 increases the frequency of spontaneous glutamatergic currents mediated by α-amino-3-hydroxy-5-methylisoxazole propionic acid (AMPA) receptors in GABAergic hippocampal interneurons [175]. Multiple types of P2X can be co-expressed in native neurons and some of them can form heteromultimers. For example, it has been shown that P2X2 and P2X3 form heteromultimers in sensory neurons [176]. In addition, P2X2 is known to have function in inflammatory and neuropathic pain, and sense of taste [147,177]. It has been suggested that stimulating P2X2 in the medial prefrontal cortex could be a potential therapeutic strategy for depressive disorders [178].

**P2X3** is a fast desensitizing receptor, almost like P2X1, and its recovery from desensitization can take longer than 15 min. It is better activated by αβmeATP or β,γ-methyleneadenosine 5′-triphosphate (βγmeATP) than by ATP [147], and the homomeric P2X3 has the lowest Ca^2+^ permeability among P2X subtypes [156]. An acidic pH inhibits P2X3, and shifts the concentration–response curve for αβmeATP to the right [179]. Potential structural reasons for the desensitizing character of this receptor was discovered directly from X-ray structures of the P2X3 receptor [180]. Further, P2X3 is exclusively expressed in primary sensory neurons [181,182], and it is known to form P2X2/P2X3 heteromeric channels [176], which play a crucial role in pain signaling [183,184,185]. In addition, ATP-activated P2X3 is also involved in taste function [177,186] or bladder contraction [187] and play a pivotal role in chronic cough [188].

**P2X4** exhibits a moderate rate of desensitization, approximately within a few seconds [147], and high Ca^2+^ permeability [156]. The first P2X structure solved was the zebrafish P2X4 crystal, and its shape was compared to a dolphin [145] (Figure 2C). Unlike other P2X, the P2X4 can be located intracellularly, specifically on lysosomal membranes, where its otherwise extracellular part is oriented into the lumen organelles [189,190]. ATP may also enter lysosomes, but the receptors cannot be activated due to inhibition at low pH [191]. Moreover, P2X4 cannot be effectively activated by αβmeATP [192], is relatively resistant to the common P2X antagonists suramin and PPADS [193], and is selectively allosterically potentiated by ivermectin, a broad-spectrum antiparasitic drug of bacterial origin [157,194]. The P2X4 subunits may assemble as functional heterotrimer with P2X6 subunit [195]. In addition, P2X4 is widely distributed throughout the CNS [4,196], and is highly expressed on microglia and macrophages [197]. P2X4 has emerged as a potential target for CNS disorders such as epilepsy, ischemia, chronic pain, anxiety, multiple sclerosis, and neurodegenerative diseases (for a review, see [198]).

**P2X5** generates low amplitude non-desensitizing currents [147]. In humans, most hP2X5 variants lack exon 10, the critical region encoding the second transmembrane domain (TM2), thereby rendering the receptor non-functional [199]. The P2X5 is highly expressed in the mouse CNS during embryonic development [200]. Further, in adult mice, it is distributed across various brain regions, including the hypothalamus [201], but its role in the brain is not well understood. For example, activation of P2X5 on satellite cells regulates skeletal muscle differentiation [202]. A study in mice P2X5 KO demonstrated the role of P2X5 in the regulation of bone loss in periodontitis [203].

**P2X6** lacks part of a binding site for ATP [204], the homomeric P2X6 is therefore non-functional and is not expressed on the cell surface [205]. However, P2X6 is thought to form functional heteromeric channels with P2X2 [206] and P2X4 [195,207]. P2X2/P2X6 heteromers are expressed in respiratory neurons of the brainstem, and recombinant P2X4/P2X6 is functional [157,195].

**P2X7** shows very low sensitivity to ATP, and no apparent desensitization when stimulated with high doses of agonists [205]. This receptor is more sensitive to 3’-O-(4-benzoyl)benzoyladenosine-5’-triphosphate (BzATP) than to ATP, exhibits high permeability for Ca^2+^, and is permeable for large molecules such as NMDG^+^, Yo-pro fluorescent dye or ethidium [147,208,209]. Within the P2X family, the P2X7 has the longest C-terminus, which is responsible for many of the specific properties of this receptor [210,211,212], including cytokine release from the brain’s resident immune cells, microglia, primed with bacterial lipopolysaccharide [186]. The P2X7 can be inhibited by low pH, bivalent cations, and Brilliant Blue-G (BBG), while being relatively insensitive to PPADS or suramin [205,208]. This is the most extensively investigated P2X subtype for drug development, and there are already many strong and selective, mainly allosteric, antagonists [213]. P2X7 is widely expressed in immune-related cells, various glial cells, Schwan cells, T-cells and macrophages [214,215]. Its activation in glial cells leads to neuroinflammation (for a review, see [216,217]), which could contribute to the development of various neurodegenerative diseases such as Alzheimer’s disease, Parkinson’s disease [218,219,220,221], and depression-related emotional disturbances, as reviewed by [222,223]. Various single nucleotide polymorphisms (SNP) have been shown to be associated with diseases and significantly influence the receptor activity by altering receptor expression and/or functional properties. For example, the *P2rx7* polymorphisms rs1718119 (Ala348Thr), rs2230912 (Gln460Arg), and rs1653625 have been shown to represent a genetic predisposition to depression. These mutations result in increased P2X7 functional responses, which may explain the pathophysiology associated with susceptibility to mood disorders [224]. Another *P2rx7* polymorphism, rs7958311, has been shown to be associated with chronic pelvic pain, fibromyalgia, and irritable bowel syndrome. This variant increase the risk of chronic overlapping pain conditions through a unique cellular profile mechanism of gain-of-function in channel current but loss-of-function in pore opening [225]. A study performed using the P2X7 reporter mice showed that the expression of the P2X7 is selectively increased in CA1 pyramidal and dentate granule neurons and microglia in mice that developed a status epilepticus after intra-amygdala injection of kainic acid [226]. After neural tissue damage or following status epilepticus, no upregulation of P2X7 protein in neurons was observed [227]. The *P2rx7*-EGFP reporter mouse, which expresses enhanced green fluorescence protein (EGFP) immediately downstream of the *P2*rx7 proximal promoter, revealed that the P2X7 is detectable in the brain of a mouse already in early embryonic development, in E9, subsequently increasing between E13 and E18, and shows specific localization in hypothalamic ependymal cells [228].

## 5. Expression and Function of P2X in Hypothalamic Nuclei

### 5.1. SON

#### 5.1.1. Expression of P2X mRNA and Protein in SON

**Figure 3:**
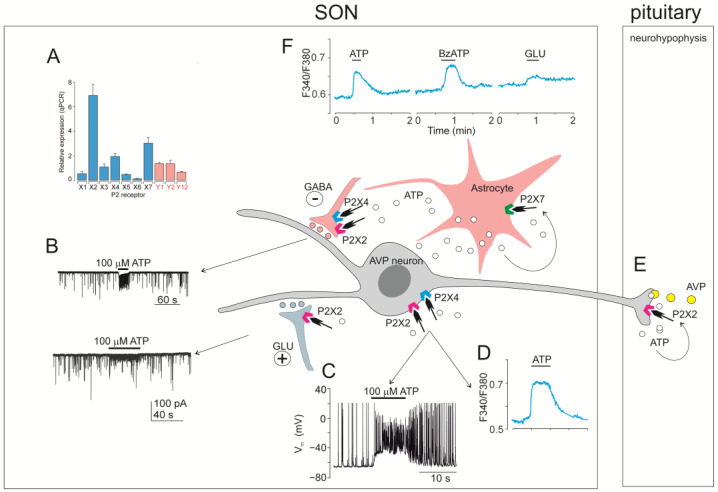
A summary of the identified mechanisms of action of ATP in magnocellular AVP neurons in the SON and posterior pituitary gland. The ATP action is based on activation of P2X2 and P2X4 receptors in somata and nerve termines of AVP neurons, and P2X7 in hypothalamic astrocytes. (**A**) Quantitative RT-PCR analysis of seven P2X (blue columns) and three P2Y (pink columns) mRNA transcripts in hypothalamic SON tissue isolated from 16 day-old rats. P2 receptor expression was related to the expression of GAPDH as a housekeeping gene [134]. (**B**) Voltage clamp whole-cell recordings of ATP-induced inward current and increase in the frequency of mIPSC (upper trace) and ATP-induced increase in the frequency of mEPSCs (lower trace). (**C**) Current clamp whole-cell recordings of action potentials before, during and after the application of ATP. (**D**) Fluorescent calcium imaging of ATP-induced response in SON neuron. (**E**) ATP release along with AVP in neurohypophysis and subsequent P2X2 stimulation. (**F**) ATP- and BzATP-induced calcium response of hypothalamic astrocyte. For experimental details, see [134].

Molecular methods proved mRNA of almost all P2X receptors in the supraoptic nucleus (SON). In situ hybridization analysis of six P2X (P2X1-P2X6) mRNAs showed that SON expresses a strong hybridization signal for three of them: P2X2, P2X4, and P2X6 [4]. Another in situ hybridization study demonstrated P2X3 and P2X4 mRNAs expression in the SON [229]. Real-time reverse-transcription (RT-PCR) analysis confirmed expression of P2X2 mRNA in SON [230]. A quantitative real-time reverse-transcription PCR (qRT-PCR) analysis of seven P2Xs (P2X1-P2X7) in circular punches of rat SON revealed the presence of P2X2, P2X3, P2X4, P2X6, and P2X7 mRNAs, with P2X3, P2X4, and P2X7 dominating [229]. Another qRT-PCR analysis of P2X1-P2X7 mRNAs and three metabotrophic P2Y mRNAs (P2Y1, P2Y2, and P2Y12) in circular punches of rat SON showed significant expressions of three of them, in the order: P2X2 > P2X7 > P2X4, moderate expression of P2X3, P2Y1, and P2Y2, and minimal expression of P2X1, P2X5, P2X6, and P2Y12 [134] (Figure 3A).

High level of P2X2 mRNA expression matched the high level of P2X2 protein in the SON. Ultrastructural studies have shown that P2X2 immunoreactivity-positive neurons and nerve fibers are localized in many hypothalamic nuclei, including those containing magnocellular neurons, such as the SON [231,232,233,234]. In the coronal sections of the rat hypothalamus (30 µm thick), the SON neuronal cell bodies were strongly P2X2 immunopositive, but only a few nerve fibers were positive [232]. Strong P2X2 immunoreactivity was also found in synapses of rat hypothalamo-neurohypophysial explant [235], which contains SON and PVN with their intact synapses and axonal projections extending through the median eminence and terminating in the posterior lobe of the pituitary gland [235]. Both, SON and the magnocellular part of PVN, displayed P2X2 labeled somata and numerous neuronal processes-axons and dendrites [235]. In addition, P2X2 immunoreactivity was observed at the axo-dendritic and axo-somatic synapses in this system [235]. Axo-dendritic synapses showed either axon terminals (synaptic buttons) marked with P2X2 and unmarked dendrites or unmarked axon terminals and marked dendrites. Moreover, axo-somatic synapses involved P2X2-positive axons on both P2X2-positive and P2X2-negative soma [235]. Heavy P2X5 immunostaining was also observed in mouse SON [201].

A double-labeling fluorescence study showed that P2Xs are differentially expressed on vasopressin- and oxytocin-containing neurons, both in SON and PVN [236,237]. Immunocytochemical labeling in isolated vasopressinergic terminals indicated the presence of P2X2, P2X3, P2X4, and P2X7, but not P2X6 [236], while P2X2, P2X3, and P2X4 were not found in terminals which labeled for oxytocin [236,238]. In the SON sections, vasopressin-containing neurons express P2X2, P2X4, P2X5, and P2X6, while oxytocin-containing neurons express P2X2, P2X4, and P2X5 [237]. Another double-labeling study revealed high colocalization of P2X5 protein and vasopressin in the SON (in 46–58% of AVP neurons) [239]. Furthermore, the distribution of immunoreactivity to P2X6 in the rat hypothalamo-neurohypophysial system studied at the electron microscope level revealed the presence of P2X6 in subpopulations of neurosecretory cell bodies, neurosecretory granules, endoplasmic reticulum, Golgi complex, neurosecretory and non-neurosecretory axons and dendrites in the SON and PVN, pituicytes and microvesicles of the neurohypophysis [240]. The authors hypothesized that P2X6 could be involved in membrane recycling of the granular vesicles and microvesicles at the neurohypophysial level [240]. In addition, immonohistochemistry indicated that P2X7 is located in both vasopressin and oxytocin terminals and soma [241,242].

#### 5.1.2. P2X Activity in SON Cells Studied by Intracellular Calcium Measurements

In the first of a number of these studies on the hypothalamus, ATP was shown to induce a rapid increase in intracellular Ca^2+^ concentration ([Ca^2+^]_i_) in non-identified fura-2M loaded rat hypothalamic neurons in culture [243]. The hydrolysis-resistant ATP analog, 2-methylthioadenosine-5′triphosphate (2MeSATP), produced a similar response, but the P2X1/P2X3 subtype agonist αβmeATP was much less effective, and ADP, adenosine-5′-monophosphate (AMP), GTP, or adenosine had no effect [243]. The ATP-induced calcium response was inhibited by nifedipine (by 62%) and by suramin, general P2X antagonist, indicating that this response resulted from the activation of P2X and influx of extracellular Ca^2+^ largely through high voltage-gated Ca^2+^ channels [243].

Consequently, calcium imaging was used to identify functional P2X in fura-2M loaded nerve terminals of magnocellular neurons isolated from posterior pituitary [244], somata of acutely enzymatically dissociated SON neurons [229], SON neurons in hypothalamo-neurohypophyseal system explants [245], and SON neurons in freshly isolated rat brain slices [134]. ATP administration increased [Ca^2+^]_i_ in approximately 40% of nerve terminals isolated from posterior pituitary, and this effect was inhibited by suramin and PPADS [244]. Locally applied ATP increased [Ca^2+^]_i_ also in somata of acutely enzymatically dissociated SON neurons [229] and SON neurons of hypothalamic slices [134]. The effects of ATP were mimicked by general purinoceptor agonists adenosine 5′-O-(2-thiotriphosphate (ATPγS) and 2MeSATP, but not by AMP, adenosine, UTP, or UDP, suggesting that P2X2 was involved [134,229]. ATP-induced [Ca^2+^]_i_ responses were prolonged in the presence of ivermectin, selective positive allosteric modulator of P2X4, suggesting the contribution of P2X4 subtype [134]. A relatively specific P2X7 agonist BzATP increased [Ca^2+^]_i_ in enzymatically dissociated SON neurons, but the potency of BzATP was lower than potency of ATP, suggesting that P2X7 was not involved [229]. In contrast, BzATP caused a more prominent increase in [Ca^2+^]_i_ than ATP in SON cells, which were considered non-neuronal because they were immunohistochemically negative for antibodies against vasopressin or oxytocin [229].

It should be emphasized here that both P2X and P2Y receptors are involved in the ATP-induced [Ca^2+^]_i_ increases observed in SON cells [134,245,246,247]. In hypothalamo-neurohypophyseal explantate, the ATP application triggered an increase in [Ca^2+^]_i_ by activating a P2Y1 subtype [245]. Also in SON slices, ATP-induced calcium responses were partially reduced (by 25%) in the presence of specific P2Y1 antagonist N(6)-methyl-2′-deoxyadenosine-3′,5′-bisphosphate (MRS2179), and the application of ADP stimulated [Ca^2+^]_i_ increase in some cells [134], most likely astrocytes [246].

#### 5.1.3. P2X Activity in SON Cells Studied by Using Electrophysiology

Extracellular recording in vivo from SON in anesthetized rats showed that locally applied ATP excites vasopressinergic neurons that were antidromically identified by means of a bipolar stimulating electrode inserted into the posterior pituitary, and distinguished from oxytocin-containing cells based on the pattern of their electrical phasic activity and their inhibition by arterial baroreceptor activation [248]. This effect was mimicked by αβmeATP and inhibited by suramin, which also blocked the excitation of SON neurons produced by vagus nerve stimulation, a procedure that excites A1 neurons and thus vasopressin neurons [248].

The intracellular recording from SON neurons in superfused explants of rat basal hypothalamus revealed that ATP and ATP agonists (αβmeATP, βγmeATP and 2MeSATP), but not adenosine, evoked depolarization that was not inhibited by tetrodotoxin (TTX), which inhibits voltage-gated sodium channels, but was reversibly inhibited by PPADS, suggesting the involvement of P2X [249].

Whole-cell patch-clamp recording from single dissociated SON neurons showed that ATP dose-dependently evoked a depolarizing inward current, which was accompanied by extracellular Ca^2+^ entry into the cell [229]. ATP-induced current, inhibited by suramin, could be recorded from both the isolated terminals and somata of magnocellular neurons in explants of the hypothalamo-neurohypophyseal system [236,238]. Electrophysiological evidence for the presence of P2X in AVP, but not in oxytocin neurons, has also been obtained, as ATP-induced current was observed in 70% of isolated vasopressin-containing neurohypophyseal nerve terminals, but not in terminals labeled for oxytocin [236]. ATP-induced somatic current was found in a subpopulation of 62% of SON neurons in acute rat brain slices [134]. The effect of ATP in slices was mimicked by 2MeSATP and ATPγS, but not by the P2X1/P2X3 agonist, αβmeATP [134]. In addition, suramin or PPADS inhibited and ivermectin and/or acid pH 6.5 potentiated the ATP-induced current, indicating the presence of both P2X2 and P2X4 in subpopulation of SON neurons, most likely AVP neurons (Figure 3) [134,250]. Finally, BzATP failed to induce membrane current in SON neurons of slices [134], while BzATP-induced current was observed in dislocated neurohypophysial terminals [242].

ATP application dramatically increases the frequency of glutamatergic miniature excitatory postsynaptic currents (mEPSCs) and GABAergic miniature inhibitory postsynaptic currents (mIPSCs) in 55% and 43% of SON neurons, respectively [134]. ATP increased the frequencies of mIPSC and mEPSC without changing their amplitude, indicating that P2X are present in presynaptic nerve terminals of neurons contacting SCN neurons and that binding of ATP to these receptors facilitated GABA release [134]. The effect of ATP on mIPSC frequency was inhibited by suramin and PPADS and potentiated by pH = 6.5, indicating involvement of presynaptic P2X2 [134]. In addition, the effect of ATP on mIPSCs frequency decreased during prolonged (40–60 s) or repeated ATP application, indicating the presence of a desensitizing P2X4 in the presynaptic membrane [134]. The application of ADP, 2MeSAMP and 2MeSADP, agonists of P2Y1 and P2Y12 receptors, failed to modulate the frequency of mIPSC [134].

#### 5.1.4. Functional Relevance of ATP Acting on P2X in the SON

Microinjection of ATP, but not ADP, AMP or adenosine, into the SON in water-loaded and ethanol-anesthetized rats decreased the outflow of urine, suggesting that ATP stimulated P2X on vasopressin-containing neurons, releasing vasopressin from nerve terminals in neurohypophysis and inducing an antidiuretic effect [251]. Also, administration of ATP to isolated neurohypophysial nerve terminals induced vasopressin release, but no significant oxytocin release, and this effect was inhibited by suramin or PPADS [236,244]. These observations are in agreement with immonohistochemical [238,241,242] and electrophysiological [134,236] evidence indicating selective expression of P2X2 and P2X4 in subpopulation of vasopressin-containing, but not in oxytocin-containing neurons.

Endogenous sources of ATP in the SON are noradrenergic A1 neurons of the caudal ventrolateral medulla [248,252,253] and astrocytes [5]. In neurohypophysis, endogenous sources of ATP are nerve terminals of magnocellular neurons, which contain ATP in vasopressin and oxytocin secretory vesicles, and release ATP together with neuropeptides [244,254,255]. Electrical field stimulation of isolated neurohypophysis caused a rapid increase in extracellular accumulation of ATP and an increase in the release of vasopressin and oxytocin [254]. However, in the presence of suramin or PPADS, only an increase in the release of vasopressin was reduced, suggesting that endogenously released ATP activated selectively P2X present in vasopressin nerve terminals and potentiated the release of vasopressin, but not oxytocin [255]. Following specific P2X knockout (KO), suramin and PPADS significantly reduce the electrically stimulated release of AVP from intact neurohypophyses of wild-type, P2X3 KO and P2X7 KO, but not P2X2/3 KO mice, suggesting that endogenous ATP stimulated AVP release via P2X2 [256].

Refeeding after fasting represents a complex stimulation to hormone secretion involving the volume/baroreceptors and peripheral/central osmoreceptors. This experimental protocol is widely used to study osmotically driven stimulation and the synthesis and release of neurohypophyseal peptides, particularly vasopressin and, to smaller extent, also oxytocin [257,258,259,260,261]. It has been found that fasting for 2 days and refeeding of rats for 2 h causes an increase in the expression of AVP and P2X2 mRNAs, but not oxytocin mRNA in the SON [250], suggesting that ATP via P2X2 likely plays a role during stimulation of vasopressin synthesis in the SON [250].

### 5.2. PVN

#### 5.2.1. Expression of P2X mRNA and Protein in PVN

In situ hybridization analysis of six P2X (P2X1-X6) mRNAs showed that paraventricular nucleus (PVN) expresses a moderate signal for P2X2, P2X4, and P2X6 [4]. Hybridization analysis of the P2X3 and P2X4 mRNA signal showed that these receptors are expressed in all regions of the PVN, i.e., in the dorsal parvocellular, medial parvocellular and posterior magnocellular component [229].

Ultrastructural studies have shown strong P2X2 and P2X4 immunoreactivity signal in the PVN [108,231,234,235,237,262,263]. P2X2 immunoreactivity was found in every subnucleus, in subpopulations of endocrine neurons, and neurosecretory and non-neurosecretory axons and dendrites [231,232], the most abundant staining was observed in the lateral parvocellular sub-division of the PVN [264]. The majority of the P2X2 immunoreactivity-positive neurons in the PVN were multipolar neurons with long axons and dendrites [232]. Axons with strong P2X2 immunoreactivity were found in the organum vasculosum of the lamina terminalis and median eminence containing nerve endings of parvocellular PVN neurons [232]. In the PVN of the rat hypothalamo-neurohypophysial explants, P2X2 immunoreactivity was found in the subpopulation of magnocellular PVN neurons and their nerve terminals in the neurohypophysis, and axo-dendritic and axo-somatic synapses [235]. Immunohistochemical analysis of the rat hypothalamo-neurohypophysial system revealed the presence of P2X6 in both neurosecretory and non-neurosecretory somata, axons and dendrites in the PVN [240].

Double-labeling fluorescence immunohistochemistry has shown that P2X are expressed differentially on magnocellular vasopressin- and oxytocin-containing PVN neurons: vasopressin-containing neurons express P2X4, P2X5, and P2X6, and oxytocin-containing neurons express only P2X4 [237]. High colocalization of P2X5 and vasopressin was reported in PVN (in 70–90% of AVP neurons) [239].

P2X2 has also been found in parvocellular CRH, TRH, and CART neurons that are associated with the regulation of food intake [108]. Injection of the retrogradely transported tracer, rhodamine-tagged microspheres, into the pressor region of the rostral ventrolateral medulla was used to identify non-neuroendocrine parvocellulr neurons in the PVN, and to determine P2X subtype on these neurons [263]. Consequently, the presence of P2X1-P2X6 subtypes was examined, and the proportions of double-labeled neurons for each of the P2X subtypes were quantified. All P2X1-P2X6 subtypes were detected throughout the PVN, and it was found that more than one subtype of P2X may be present on a single neuron, with the P2X3 and P2X5 being dominant [263]. In addition, double-immunofluorescence showed that P2X7 is co-localized with the microglial marker in the PVN [265].

#### 5.2.2. P2X Activity in Magnocellular PVN Neurons Studied by Using Electrophysiology

Whole-cell patch-clamp recordings obtained from rat magnocellular PVN neurons, which were identified by their distinct morphology and electrophysiological characteristics, showed that ATP failed to evoke somatic currents [135]. Application of norepinephrine or ATP or BzATP induced a sustained increase in the mEPSC amplitude, and the effect of norepinephrine was inhibited by BBG, the P2X7 antagonist [135]. Interestingly, some studies provided evidence that P2X7 can directly activate phosphatidylinositol-3-kinase (PI3K), a key mediator of AMPA receptor insertion into the cell membrane at hypothalamic glutamate synapses [266,267,268]. Therefore, it has been suggested that endogenously released ATP regulates synaptic plasticity in PVN through the increase in postsynaptic calcium, and the insertion of new AMPA receptors into the postsynaptic membrane [135,268].

Another whole-cell patch-clamp recording obtained from magnocellular PVN neurons, identified by their anatomical location in rat brain slices and their unique electrophysiological properties during depolarizing current injection, showed that application of ATP increased the frequency of current-stimulated action potentials [269]. This effect of ATP was blocked by either PPADS and/or kynurenic acid (a glutamate receptor antagonist) [269]. It has been suggested that ATP triggered a shift in the holding current, which was caused by an ATP-induced increase in a tonic extrasynaptic glutamatergic excitatory current, [269]. As the hyperosmotic stimulus also exhibits potentiating effect on N-methyl-D-aspartate (NMDA)-evoked currents and increases the frequency of action potentials in magnocellular neurons, it has been suggested that there is a functional excitatory coupling between P2 purinoreceptors and extrasynaptic N-methyl-D-aspartate (NMDA) receptors [269].

#### 5.2.3. P2X Activity in Parvocellular PVN Neurons Studied by Using Electrophysiology

The presence of functional P2X2 on parvocellular PVN neurons was confirmed by single-channel recordings, obtained from outside-out patches in coronal whole-brain slices [270]. Analyses of the single-channel properties of native P2X have shown that at least two distinct P2X populations are present. The first showing “flickery” openings and the second showing “non-flickery” openings. These populations are likely to consist of mixtures of homomeric and heteromeric P2X2, possibly containing P2X2, P2X3, P2X4, and P2X6 subunits [270].

Whole-cell patch-clamp recordings from retrogradely labeled parvocellular neurons projecting to the rostral ventrolateral medulla in the brainstem region showed that ATP application increases the frequency of action potentials in identified neurons, and that this effect is blocked by PPADS and/or kynurenic acid [271]. ATP had no significant effect on the frequency or amplitude of spontaneous excitatory postsynaptic currents (sEPSC), but increased the amplitude of current induced by focal application of AMPA. This ATP effect was blocked by PPADS and chelation of intracellular Ca^2+^ with BAPTA [271]. Further, hyperosmotic stimulus potentiated the AMPA-evoked currents, and this effect was also blocked by PPADS [271]. Thus, it has been suggested that there is a functional stimulatory coupling between extrasynaptically located P2 purinoreceptors and AMPA receptors, which plays a role in the response of parvocellular PVN neurons to acute hyperosmotic stimulus and which in turn could contribute to osmotically driven sympathoexcitatory reactions of PVN [271].

Another whole-cell patch-clamp recording was performed on identified PVN neurons in slices isolated from transgenic mice expressing tdTomato under the control of the P2X4 gene (*P2rx4* tdTomato mice). These recordings showed that ATP application does not evoke inward currents that would indicate the presence of functional P2X4 in somata, but increased the frequency and amplitude of mIPSC in some PVN neurons [272]. The ATP-evoked increase in mIPSC frequency was due to the activation of presynaptic P2X4 expressed in the terminals of AgRP/NPY neurons originating from arcuate nucleus (ARC) and projecting to the PVN. These experiments showed that presynaptic P2X4 regulates the release of GABA onto parvocellular PVN neurons involved in sympathoexcitation [272].

#### 5.2.4. Functional Relevance of ATP Acting at P2X in the PVN

Microinjection of ATP into the PVN of water-loaded and ethanol-anesthetized rats produced an antidiuretic effect, while ADP, AMP and adenosine reduced the outflow of urine much less than ATP [273]. The antidiuretic effect of ATP was blocked by prior injection of quinidine (a P2-purinoceptor antagonist) into the PVN, suggesting that ATP activated P2X on magnocellular vasopressin-containing neurons, releasing AVP in the posterior pituitary gland and stimulating AVP(V2) receptors in the kidney [273].

Microinjection of PPADS into the PVN increased blood pressure and heart rate [274,275], indicating that P2X are also involved in the modulation of autonomic function that control the cardiovascular system [274]. Microinjection of αβmeATP [276] or ATP [277] to PVN in vivo and/or in decorticated, unanesthetized, arterially perfused in situ preparation of rat caused pressor and tachycardiac responses [276,277]. Cardiovascular responses evoked by microinjection of αβmeATP or ATP were reduced by pretreatment with PPADS or the glutamate receptor antagonists [276,277], suggesting that ATP-mediated sympatho-excitation depends, at least in part, on the activation of ionotropic glutamate receptors and coupling between extrasynaptically located P2 purinoreceptors and AMPA receptors [271].

Salt-loading, in which drinking water is replaced with 2% NaCl solution for up to 4 days, is an experimental model known to cause a gradual increase in blood pressure and plasma osmolarity that activates oxytocin and vasopressin neurons to a similar extent, causing depletion of stores of both hormones in pituitary [43]. There are several mechanisms, by which salt-loading could influence the activity of vasopressin neurons [278,279] These include potentiation of glutamatergic synaptic inputs observed in the SON [27], or norepinephrine-induced release of ATP from astrocytes that are positioned in close proximity of synapses to sense and modulate the afferent synaptic activity described in the PVN [135]. For example, immunohistochemical assessment of glial-fibrillary acidic protein (GFAP) revealed that salt-loading increased glial cell reactivity in the PVN, and measurements of extracellular ATP and adenosine accumulation via biosensors in hypothalamic slices showed that baseline ATP release was increased 17-fold in the PVN, while adenosine remained unchanged. Furthermore, disruption of Ca^2+^-dependent vesicular release mechanisms in PVN by virally driven expression of a dominant-negative SNARE protein decreases the release of ATP, indicating that salt-loading stimulates the release of ATP in the PVN from glial cells through the mechanism of Ca^2+^-dependent exocytosis. It indicates that astrocytic ATP likely contributes to the neural control of blood pressure and plasma osmolarity [280].

Systemic osmotic stimulation induced by intravenous administration of hypertonic solutions triggers a rapid neurohumoral response, which includes release of vasopressin from the posterior pituitary, activation of the sympathetic nervous system, and increases in blood pressure. It was observed that hyperosmotic stimulation increased the number of activated (Fos-positive) P2X2-expressing PVN neurons, and this effect was reduced after PPADS pre-treatment, indicating that a subset of P2X2 expressing PVN neurons might facilitate increased sympathetic activity [264].

Glial-derived ATP also contributes to the effect of ghrelin on vasopressin neurons in the PVN of rat brain slices [281]. Activation of P2X7 in PVN microglia mediating the production of proinflammatory cytokines resulted in increased activity of oxytocinergic and vasopressinergic neurons and activity of the renal sympathetic nervous system [265].

The endogenous source of ATP in the PVN is also blood [282]. PVN is characterized by high capillary density, thin blood vessels, and complex vascular topology compared to other areas of the brain. The PVN is thus susceptible to penetration of ATP released from vasculature in response to hemodynamic disturbance after blood pressure increase, which promotes sympathetic overexcitation in hypertension [282].

### 5.3. SCN

#### 5.3.1. Expression of P2X mRNA and Protein in the SCN

Strong signal for P2X2, P2X4, and P2X6 mRNA was found in the SCN using in situ hybridization [4,283]. Expression of seven P2Xs (P2X1-7) was studied in circular punches of rat SCN using qRT-PCR [233]. Transcripts for all P2X subunits were found in the SCN, and the relative expression levels of mRNA corresponded to the order: P2X2 > P2X7 > P2X4 > P2X5 > P2X1 = P2X3; the amount of the P2X6 mRNA was very low [233]. Compared to the SON which was examined in the same way in the same laboratory [134], the amount of P2X2 mRNA in the SCN was much lower, about half [233]. Next, spatial and time-of-day-dependent variations in expression of seven P2Xs (P2X1-7) were analyzed in mouse SCN using qRT-PCR and immunohistochemistry [234]. The relative expression levels of mRNA followed the order: P2X7 > P2X3 > P2X4 > P2X2 > P2X5 > P2X1 > P2X6. Among seven P2X receptors, the following five showed a time-of-day-dependent variation in the relative expression of mRNA: P2X2, P2X3, P2X4, P2X5, and P2X7. All of these rhythmically expressed receptors showed minimal levels of expression at the end of the dark phase (ZT22) and peak levels of expression at the early dark phase (ZT14) [234].

Quantitative immunohistochemical analysis showed a moderate P2X2, P2X4, P2X5, and P2X7 immunoreactivity and very weak P2X1, P2X3, and P2X6 immunoreactivity in the bodies and fibers of SCN neurons [234]. P2X4 immunoreactivity rises in neuronal somata during the dark phase (at ZT 14–22), especially in the ventrolateral region of the SCN, while P2X2 immunoreactivity displays no time-of-day-dependent variation [234]. Interestingly, this finding was not specific to SCN, and time-of-day-dependent P2 receptor expression was also found in various hippocampal layers [284]. Immunohistochemical staining for P2X2 performed in rat brain coronal sections showed that P2X2 immunoreactivity was absent on the cell bodies of SCN neurons, but confocal microscopy revealed the colocalization of P2X2 with the presynaptic marker of synapsin in numerous, but not all, nerve terminals of SCN neurons in primary cell culture [233]. In addition, P2X2 immunoreactivity-positive neurons and nerve fibers were found in the retrochiasmatic area [232], a region located behind the SCN and in front of arcuate nucleus. This area receives projections from the SCN, and sends outputs to control the daily production of melatonin in the pineal gland [285].

Interestingly, a double-labeling fluorescence study revealed that, unlike SON and PVN, there is no collocalization of P2X5 and vasopressin in the SCN [239]. Another double-labeling study showed a relatively high signal of P2X7 colocalized with glial-fibrillary acidic protein (GFAP) throughout the SCN region in acutely isolated rat brain slices [6], and experiments performed on the *P2rx7*-EGFP reporter mouse confirmed positive signal for P2X7 in the SCN [228].

#### 5.3.2. P2X Activity in SCN Cells Studied by Intracellular Calcium Measurements

Single cell calcium measurements in Fura-2AM loaded hypothalamic slices showed that ATP application induces [Ca^2+^]_i_ increase in 44% of SCN cells, while application of BzATP, ADP, or αβme-ATP induced an increase in [Ca^2+^]_i_ in only 28%, 26%, 12%, and 11% of SCN cells, respectively [233]. ATP-induced [Ca^2+^]_i_ increases were inhibited by non-selective P2X/P2Y receptor antagonists suramin and PPADS, and partly by tetrodotoxin (TTX), indicating that activation of P2X in neurons contributed to the ATP-stimulated [Ca^2+^]_i_ responses in SCN slices [233]. Additionally, in the primary culture of SCN astrocytes, the ATP- and BzATP-stimulated [Ca^2+^]_i_ responses were inhibited by blockers of P2X7 (AZ 10606120 and A438079) and reduced by MRS2179, blocker of P2Y1, indicating that the ATP-stimulated [Ca^2+^]_i_ responses in SCN astrocytes resulted from activation of P2X7 and P2Y receptors [6].

#### 5.3.3. P2X Activity in SCN Neurons Studied by Using Electrophysiology

Whole-cell patch-clamp recordings from SCN neurons in rat brain slices revealed that ATP application induced very small somatic current in just 7% of ventrolateral neurons producing vasoactive intestinal peptide (VIP), and no current in endogenously rhythmic neurons in the dorsomedial region producing AVP [233]. However, ATP application caused a dramatic increase in the frequency mIPSC, without changing their amplitude, in 40% of SCN neurons, both in the ventrolateral and dorsomedial regions, indicating that P2X are present in the nerve terminals of presynaptic neurons contacting SCN neurons [233]. In addition, the ATP-induced increase in mIPSC frequency was inhibited by PPADS and mimicked by 2MeS-ATP or ATPγS, but not by BzATP or ADP, suggesting that presynaptic neurons in the SCN express P2X2 [233].

#### 5.3.4. Functional Relevance of ATP Acting at P2X in the SCN

The ATP content and extracellular ATP concentrations in the SCN show a time-of-day-dependent rhythm that negatively correlates with the rhythm of electrical and metabolic activity of SCN neurons, i.e., the extracellular ATP is high at night and low during the day [6,286,287]. The circadian rhythm in extracellular ATP accumulation matches the circadian rhythm of glia activity, which is also in antiphase with the daily rhythm of electrical activity of SCN neurons [288,289], indicating that ATP is released from SCN astrocytes [6], and the rhythm of ATP secretion is controlled by clock genes in astrocytes [290].

Furthermore, SCN2.2 cells, an SCN-derived cell culture containing 80% astrocytes, generate circadian oscillations in both the production and extracellular accumulation of ATP with an average period of 23.7 h [287,291]. Extracellular accumulation of ATP in SCN organotypic cultures depends on the time of day, peaking between 24:00 and 04:00 and minimal at ∼12:00 [6]. Several selective P2X7 antagonists (AZ10606120, A438079, and BBG) have been shown to inhibit extracellular ATP accumulation in organotypic cultures of SCN [6], suggesting that ATP may go out of the cell via P2X7 channels, and that extracellular ATP allows astrocytes to fine-tune their activity with autocrine/paracrine signaling via P2X7.

Interestingly, ATP levels (measured as ATP content in 2 mm-thick coronal slices) have been reported to increase in the initial hours of spontaneous sleep in wake-active regions (i.e., basal forebrain and lateral hypothalamus), but not in sleep-active regions (i.e., ventrolateral preoptic region) of the rat [292]. This ATP increase depends on sleep, but not on the time of day, suggesting that the circadian rhythm of extracellular ATP accumulation in the SCN does not appear to be related to sleep, but rather represents an extracellular signal that could, for example, synchronize SCN neurons. Further studies are needed to uncover the role of extracellular ATP accumulation in the SCN.

### 5.4. AVPV

#### 5.4.1. Expression of P2X mRNA and Protein in GnRH Neurons of AVPV

In situ hybridization showed that the median preoptic nucleus (MnPO), an area that belongs to the preoptic region (POA) and is located near the third ventricle, like the anteroventral periventricular nucleus (AVPV), expresses a strong signal for P2X4 mRNA, a moderate signal for P2X6 mRNA, but the signal for P2X2 mRNA is absent [293].

GnRH neurons originate primarily from embryonic olfactory placode and migrate to specific regions in the brain during development. RT-PCR analysis identified P2X2 and P2X4 in embryonic GnRH neurons in culture from olfactory placode of rhesus monkeys [294]. In adult animals, the distribution of GnRH-positive neurons is diffuse in the brain, hindering efforts to monitor their P2X expression at the tissue level. A detailed analysis was therefore performed on the transcriptome of an entire set of GnRH neurons obtained from coronal brain section of intact, proestrous, and metestrous female GnRH-GFP transgenic mice [295]. About 1500 individual GnRH neurons were sampled from proestrous and metestrous groups, their transcriptome was analyzed using microarray hybridization and real-time PCR, and changes in mRNA expression of genes involved in neurotransmitter signaling during the estrous cycle were investigated. Among P2X, differential gene expression during the estrous cycle was most evident in *P2rx7* gene, whose expression was significantly down-regulated by estrogen in proestrous GnRH neurons, suggesting that P2X7 contributes to the induction of the pre-ovulatory GnRH surge [295].

Immunohistochemical analysis showed that P2X2 immunoreactivity-positive neurons and nerve fibers [108,232], and ectonucleotide pyrophosphatase/phosphodiesterase 1 (NPP1) [296] are abundantly expressed in the periventricular nucleus (PeN). Double-labeling fluorescence immunohistochemistry examined colocalization between GnRH and P2X1–6 receptors in mice hypothalamus [78]. This study showed that P2X2, P2X4, P2X5, and P2X6, but not P2X1 and P2X3, which are expressed on the perikarya of GnRH neurons in the region around the organum vasculosum of the lamina terminalis (OVLT), and P2X2 and P2X6 are expressed on the axon terminals of GnRH neurons in the median eminence [78].

#### 5.4.2. Expression of P2X mRNA and Protein in Kisspeptin Neurons of AVPV

RNA sequencing (RNA-seq) analysis performed on selectively isolated and actively translated mRNAs from just AVPV *Kiss1* neurons of female mice showed that the P2X5 receptor gene (*P2rx5)* is expressed in AVPV kisspeptin neurons and that estrogen upregulated *P2rx5* expression [76], suggesting that ATP-activated P2X5 signaling in AVPV kisspeptin neurons may be involved in GnRH/LH surge generation [76]. Another RNA-seq analysis revealed that the P2X2 receptor gene (*P2rx2*) is highly expressed in AVPV *Kiss1* neurons of rats, and is upregulated by estrogen [297]. However, this study failed to detect the expression of other purinergic receptor genes regardless of estrogen milieu, indicating that there may be a species difference in the type of P2X in AVPV *kisspeptin* neurons [297].

Immunohistochemical analysis showed that the proestrous level of estrogen significantly increased the number of P2X2-immunopositive AVPV *kisspeptin* neurons, visualized by tdTomato in *Kiss1*-tdTomato rats, suggesting that ATP-purinergic signaling is involved in estrogen-dependent regulation of reproduction [297].

#### 5.4.3. P2X Activity in GnRH and Kisspeptin Neurons in AVPV Studied by Intracellular Calcium Measurements

Application of ATP, ATP-γS and 2MeSATP to GnRH neurons in fura-2 AM loaded culture derived from the olfactory placode stimulated an increase in [Ca^2+^]_i_, while the response to αβmeATP was significantly smaller, and ADP, AMP, or BzATP elicited no response [294]. ATP-induced [Ca^2+^]_i_ response was blocked by PPADS, indicating that functional P2X2 or P2X4 are present in embryonal GnRH neurons [294]. ATP also stimulated [Ca^2+^]_i_ increases in non-neuronal cells of the olfactory culture, and these cells often responded with a small amplitude to ADP and AMP, but not to adenosine, indicating the presence of P2Y [294]. Cells in olfactory placode culture exhibit spontaneous [Ca^2+^]_i_ oscillations that are supposed to be involved in synchronization between GnRH neurons and non-neuronal cells [294]. Inhibition of P2X by PPADS has no effect on spontaneous [Ca^2+^]_i_ oscillations in cultured GnRH neurons from nasal explants of mouse embryos [298].

Intracellular calcium measurements in the immortalized cell line of GnRH neurons (GT1 cells), which have neuronal markers and secrete GnRH [299], did not confirm endogenous expression of any functional P2 purinergic receptor (P2X or P2Y) [300]. By contrast, functional P2X2 was identified in immortalized cell line of AVPV kisspeptin neurons (mHypoA-51 cells), in which ATP application increased [Ca^2+^]_i_ and co-administration of PPADS blocked this effect [297].

#### 5.4.4. P2X Activity in GnRH and Kisspeptin Neurons Studied by Using Electrophysiology

Although numerous neurotransmitters and gliotransmitters have been shown to directly modulate the electrical activity of GnRH neurons and kisspeptin neurons (for a review, see [77]), no direct effects of extracellular ATP are described yet.

#### 5.4.5. Functional Relevance of ATP Acting at P2X in the AVPV

Infusion of ATP, and not ADP or AMP, over olfactory cultures from the rhesus monkey embryo stimulated the release of GnRH [294]. The application of ATP, ADP, and αβ-methylene ATP, but not AMP or adenosine, stimulated GnRH release also from explants of median eminence [301]. Furthermore, inhibition on E-NTPDases in the neuroendocrine hypothalamus increased the midcycle LH surge, suggesting that elevated endogenous ATP concentration facilitates GnRH neuron secretory activity by activating P2X receptors [302]. Administration of ATP into the AVPV region in estrogen-treated ovariectomized rats stimulated a surge-like increase in LH release, but could not increase LH release in *Kiss1* KO rats, suggesting that ATP triggers GnRH/LH surge by activating P2X expressed selectively on kisspeptin neurons [297]. Since kisspeptin neurons, but not GnRH neurons, express also estrogen receptors, kisspeptin neurons are primarily responsible for the estrogen-dependent upregulation of P2X expression observed in mice [76] and rats [297].

Immortalized mouse GnRH neurons spontaneously release ATP in culture [303], and ATP is also released along with GnRH from nerve terminals in the median eminence into the hypophyseal-portal system vasculature in ovariectomized sheep [304]. Therefore, elevated concentrations of endogenous ATP in the hypophyseal-portal system may facilitate LH release at the level of pituitary gonadotrophs [305,306], which selectively express P2X2 [307].

### 5.5. AHN

#### 5.5.1. Expression of P2X in the AHN

While extracellular ATP has been found to be involved in the development of fever [308], there is no evidence of P2X expression in AHN warm-sensitive neurons.

#### 5.5.2. Functional Relevance of ATP in the AHN

At the level of the anterior hypothalamus, fever is initiated by interleukin IL-1beta [309,310]. There is much evidence showing that extracellular ATP stimulates the release of cytokines, such as interleukin IL-1beta and tumor necrosis factor [311,312]. In human monocytes, this effect of ATP may be completely blocked by the P2X7 antagonist, oxidized ATP (oATP), and partly by the antibody against P2X7 [311], suggesting that P2X7 and changes in the level of extracellular ATP in the AHN play a role in the development of the febrile response [308].

The possible involvement of P2X in the central mechanisms of body temperature regulation was studied by injecting ATP into the third ventricle in conscious rats at various ambient temperatures and during a fever induced by bacterial endotoxin, lipopolysaccharide (LPS) [308]. Intracerebroventricular injection of αβmeATP, a stable ATP analog, induced a decrease or increase in body temperature depending on ambient temperature, and this effect was regulated by general P2X antagonists suramin and PPADS [308], suggesting that the population of warm-sensitive AHN neurons could be the site of action of extracellular ATP [310]. Systemic application of Brilliant Blue G (BBG), specific inhibitor of P2X7, attenuated both fever and increases in plasma cytokine levels evoked by LPS in rats [313].

Next, ATP release was measured in anterior hypothalamus of conscious rabbits in real time with amperometric biosensors during systemic inflammation induced by intravenous injection of LPS. An increase in ATP concentration was observed, confirming that ATP release plays a crucial role in the development of thermoregulatory febrile response [314]. However, all animals treated with BBG, PPADS, or oATP application into the anterior hypothalamus remained febrile 6 h after induction of systemic inflammation by intravenous administration of LPS [314].

### 5.6. ARC

#### 5.6.1. Expression of P2X mRNA and Protein in ARC

In situ hybridization revealed that ARC expresses a strong signal for P2X2 mRNA, and moderate signals for P2X4 and P2X6 mRNAs [4]. After that, RT-PCR analysis confirmed expression of P2X2 mRNA in ARC [283]. Finally, RNA-seq analysis revealed that, unlike AVPV kisspeptin neurons, *P2rx2* is not expressed in ARC kisspeptin neurons [297].

P2X2 immunostaining showed similar labeling of cell bodies and fibers in the ARC as described in the PVN and SON [231,232,262,283]. Particularly strong P2X2 immunoreactivity was found in the cellular bodies of orexigenic AgRP/NPY neurons and neural extensions located in the ventromedial part of the ARC, a subregion of the nucleus with a weak blood–brain barrier, involved in the regulation of food intake [108]. Furthermore, a double-labeling showed that P2X2 immunoreactivity was only occasionally detected on anorexigenic POMC/CART neurons and α-MSH- or DYN-containing neurons [108]. Neurons synthesizing somatostatin or nNOS do not appear to express P2X2 [108]. Additionally, immunohistochemistry and genetic crosses of *P2rx4* tdTomato reporter mice with cell-type-specific GFP reporter lines showed that the tdTomato-expressing cells are primarily AgRP/NPY neurons and tanycytes, providing evidence that P2X4 is expressed in these cells [272]. Finally, P2X7 was not found to be located in the ARC of *P2rx7*-EGFP reporter mouse [228].

It should be noted here that P2Y1-immunopositive cells were also detected in rat ARC, and their expression was modified by reduced food availability [315]. AgRP neurons also express P2Y6 [316].

#### 5.6.2. P2X Activity in ARC Cells Studied by Intracellular Calcium Measurements

Application of ATP and 2-MeSATP, but not the P2X1/P2X3 agonist αβmeATP, induced [Ca^2+^]_i_ increase in the primary neuroglial cell culture of rat ARC [317]. In the absence of extracellular calcium, the 2-MeSATP-stimulated [Ca^2+^]_i_ increase was suppressed in all ARC neurons, but only in 25% of astrocytes. Highly P2Y1-selective agonists, MRS2365 and 2-methylthioadenosine-5′diphosphate (2-MeSADP), activated 75–85% of all ARC astrocytes responding to 2-MeSATP [317]. It means that ARC neurons express P2X and the majority of ARC astrocytes express P2Y.

#### 5.6.3. P2X Activity in ARC Cells Studied Using Electrophysiology

Whole-cell patch-clamp recording from single dissociated ARC neurons showed that the application of ATP evokes a large inward current that was inhibited by PPADS [318]. Also ATPγS and 2-MeSATP, but not αβmeATP or βγmeATP, evoked inward currents, suggesting that ARC neurons express functional P2X2 and/or P2X4 [318]. No evidence was found for ATP-evoked inward currents that would be indicative of the presence of functional P2X on somata of identified AgRP/NPY neurons in slices of either wild-type or *P2rx4* tdTomato mice [272]. However, ATP application dramatically increased the frequency, but not the amplitude, of mIPSC in POMC neurons contacted by AgRP/NPY neurons, indicating that facilitation of spontaneous GABA release was caused by activation of presynaptic P2X4 in nerve terminals of AgRP/NPY neurons [272].

Perforated whole-cell patch-clamp recording showed that activation of P2Y6 by UDP increased the firing rate of AgRP neurons in lean mice and subsequently promoted feeding [319].

#### 5.6.4. Role of ATP Acting in ARC Physiology

Food-restriction for 3 or 10 days and refeeding of rats after a restriction revealed enhanced expression of hypothalamic P2Y1 and nNOS mRNAs in cell bodies and cellular processes of ARC neurons and astrocytes, indicating that restricted feeding may enhance the sensitivity of the hypothalamus to extracellular ADP/ATP by regulation of the expression of P2Y1 [315]. It has also been observed that hypothalamic UDP contents increases in obese and diabetic mice [319], and that inhibition of P2Y6 signaling in AgRP neurons reduces food intake and fat mass [316]. In addition, Khakh et al. did not find any evidence that ATP regulates feeding behavior, either when ATP was applied within the ARC in vivo or when P2X4 receptors were genetically deleted [272].

### 5.7. VMH

#### 5.7.1. Expression of P2X mRNA and Protein in the VMH

In situ hybridization study revealed a strong signal for P2X2 mRNA and moderate signals for P2X4 and P2X6 mRNAs in the VMH [4]. RT-PCR analysis confirmed expression of P2X2 mRNA in VMH [283]. Immunoreactivity studies showed no or low number of P2X2-positive neurons in the VMH [231,232]. Experiments performed on hypothalamic slices of transgenic mice selectively expressing enhanced GFP in SF-1-positive neurons (SF-1-GFP-positive neurons, characterizing VMH) revealed that 51% of these neurons express P2X4, while only 3% express P2X2 [98]. Moreover, P2X2 and P2X4 receptors are also expressed by non-SF-1-GFP-positive neurons in VMH [98]. The intensity of P2X5 immunofluorescence in VMH is low [239]. In addition, a P2rx7-EGFP reporter mouse was found to have a positive P2X7 signal in the nearby ependymal hypothalamic region, but it was not located in VHM itself [228].

#### 5.7.2. P2X Activity in VMH Cells Studied by Intracellular Calcium Measurements

ATP application induced [Ca^2+^]_i_ increase in acutely dissociated VMH neurons of the rat [320]. The effect of ATP was mimicked by 2MeSATP and ATPγS, potentiated by Zn^2+^ and inhibited by suramin and PPADS [320]. ATP induced only a small [Ca^2+^]_i_ increase when extracellular Na^+^ is removed, even in the presence of 10 mM Ca^2+^, suggesting that the [Ca^2+^]_i_ rise occurred primarily as a result of membrane depolarization and activation of voltage-dependent Ca^2+^ channels, rather than by an influx via the P2X channel [320].

#### 5.7.3. P2X Activity in VMH Cells Studied by Using Electrophysiology

Whole-cell patch-clamp recording from acutely dissociated VMH neurons of the rat showed that application of ATP evoked a large inward current indicating that ATP activated ionotropic P2X receptors [320]. Patch-clamp recording from SF-1-positive VMH neurons in slices from transgenic mice showed that application of ATP increases frequency of action potentials, but induces inward current of small amplitude [98], which may be due to low number of somatic P2X2 and/or expression of desensitizing P2X4 in these neurons.

Increased surface expression of P2X4, triggered by the use of a peptide competing with P2X4 intracellular endocytosis motif, decreased the frequency and amplitude of GABAergic postsynaptic currents and increased the excitability of SF-1-GFP-positive neurons [98]. Interestingly, co-immunopurification and pull down assays revealed that P2X4 makes a complex with GABA_A_ receptor, suggesting that the formation of the P2X4/GABA_A_ complex at postsynaptic sites may regulate GABAergic synaptic input and neuronal output in the VMH [98].

#### 5.7.4. Functional Role of ATP Acting at P2 Receptors in the VMH

Intracerebroventricular infusion of ATP/ADP analogs 2-MeSATP and adenosine 5′-O-(2-thiodiphosphate (ADPβS) increased food intake in 18 h food-deprived rats [321]. This effect was abolished by pretreatment with the non-selective P2X/P2Y antagonist PPADS or selective P2Y1 antagonist MRS2179, suggesting that ATP/ADP-induced regulation of food intake is dependent on P2Y1 activation [321]. P2Y1 has also been reported to be involved in obesity [322], and P2Y1 shows major expression in hypothalamic astrocytes of Zucker diabetic fatty (ZDF) rats, which innately develop obesity associated with increases expression of hypothalamic P2Y1 [323].

### 5.8. DMH

#### 5.8.1. Expression of P2X mRNA and Protein in DMH

Available information on specific expression of P2X mRNA or protein in the dorsomedial nucleus of the hypothalamus (DMH) are still missing. On the other hand, the co-localization of P2Y1 and nNOS was demonstrated on some neurons in this region [315].

#### 5.8.2. P2X Activity in DMH Cells Studied by Intracellular Calcium Measurements

Application of ATP, 2-MeS-ATP, or ATPgS stimulated the rise in [Ca^2+^]_i_ in neurons mechanically dissociated from slices containing DMH region [324]. This effect was reduced by suramin and PPADS, and similar potency of ATP, 2-MeS-ATP, and ATPgS is consistent with the pharmacological profile of P2X2 subtypes [324]. The ATP-induced increase in [Ca^2+^]_i_ was dependent on external Ca^2+^ and Na^+^, and was substantially inhibited by voltage-dependent Ca^2+^ channel blockers, meaning that the [Ca^2+^]_i_ increase occurred primarily due to membrane depolarization and activation of voltage-dependent Ca^2+^ channels [324].

#### 5.8.3. P2X Activity in DMH Cells Studied by Using Electrophysiology

Perforated whole-cell patch-clamp recording performed on mechanically dissociated DMH neurons of the rat showed that application of ATP induced an inward current [324]. Rapid rise-time and slow desensitization kinetics was consistent with the expression of functional P2X2 [324]. Furthermore, whole-cell patch-clamp recordings of evoked IPSCs from DMH neurons in rat brain slices showed that ATPγS, a non-hydrolyzable analog of ATP, increases the amplitude of IPSC and potentiates GABA release from inhibitory nerve terminals through activation of presynaptic P2X [124]. It has been suggested that the activation of neighboring astrocytes could trigger the release of a gliotransmitter (ATP) that acts on the presynaptic terminal to stimulate GABA release in the GABA synapses in the DMH [124].

#### 5.8.4. Functional Relevance of ATP Acting on P2 Receptors in the DMH

The mechanisms involving activation of presynaptic P2X and increase in GABA-ergic transmission under basal conditions and following high frequency stimulation could potentially represent modulation of inhibition of orexigenic neurons in DMH, thus influencing feeding behavior [124]. However, the functional relevance of presynaptic P2X in DMH has not yet been explained in detail.

It has also been suggested that extracellular ATP in DMH is involved in modulating fear and anxiety behavior in the rat [325]. Possible involvement of P2 receptors has been studied by investigating the influences of P2X1/P2X3 agonist αβmeATP, the unspecific P2 receptor antagonist PPADS, the P2Y1-, P2Y11-, and P2Y12-specific agonist adenosine 5′-O-(2-thiodiphosphate) (ADPbetaS), and the specific P2Y1 antagonist MRS 2179 on rat behavior in an elevated plus-maze [325]. The anxiolytic-like effects were observed after activation of P2Y1, and there was evidence of co-localization of P2Y1 and neuronal NOS (nNOS) on some neurons in this region, suggesting that P2Y1 is involved in anxiety modulation in rats in close association with related nitric oxide production in DMH [325].

### 5.9. TMN

#### 5.9.1. Expression of P2X mRNA and Protein in TMN

In situ hybridization showed that TMN expresses a strong signal for P2X4 and P2X6 mRNAs, but the intensity of hybridization signal of P2X2 mRNA was very low [4]. However, RT-PCR analysis showed expression of P2X2 mRNA in TMN [283]. Single-cell RT–PCR (scRT–PCR) analysis revealed that P2X2 mRNA is expressed in the majority of histaminergic neurons in TMN, while each of P2X1, P2X3, P2X4, P2X5, and P2X6 mRNAs was detected in less than 35% of the cells [326]. Single-cell RT-PCR also revealed that most of the histaminergic neurons in TMN express more than one subtype of P2X on soma [327].

Ultrastructural studies have shown the presence of P2X2 immunoreactivity-positive neurons and nerve fibers in TMN [231,262]. A detailed analysis of P2X2 distribution in TMN revealed that neuronal cell bodies are strongly positive, but few nerve fibers were P2X2-positive [232].

#### 5.9.2. P2X Activity in TMN Cells Studied by Electrophysiology

Slowly desensitizing P2X-mediated ATP-evoked currents have been recorded in neurons freshly dissociated from the TMN using the nystatin-perforated patch clamp technique [328]. The potency of ATP analogs was in the order of ATP ≥ 2MeSATP >> αβmeATP. Neither ADP, adenosine nor AMP induced any response, suggesting that TMN neurons express functional somatic P2X2 [328]. In addition, the ATP-induced current recorded from histaminergic neurons of the TMN was potentiated by acidification of the extracellular solution (pH = 6.6) and inhibited by suramin and PPADS, Cibacron blue, and trinitrophenyl adenosine 5′-triphosphate (TNP), suggesting that P2X2 is the major purinoreceptor on the soma of TMN neurons [326,327].

#### 5.9.3. Functional Relevance of ATP Acting at P2X in the TMN

TMN is an important histaminergic nucleus in the hypothalamus involved in arousal and wakefulness, but to our knowledge, the functional significance of extracellular ATP and P2X in this region has not yet been studied. Since the homeostatic theory of sleep involves ATP depletion and adenosine accumulation in the brain [292], the role of extracellular ATP and P2X receptors in major sleep-controlling centers, such as TMN, should be examined.

### 5.10. LHA

#### 5.10.1. Expression of P2X mRNA and Protein in the LHA

Although direct studies on LHA P2X mRNA expression are lacking, immunohistochemical analysis showed a specific localizations of P2X2 at orexinergic neurons in the lateral hypothalamus [329]. In some LHA cells, P2X2 was co-localized with dynorphin peptides (DYN) [108], which are the endogenous ligands for the kappa opioid receptors (KOR), that regulate food intake [109]. Furthermore, double immunohistochemistry revealed that virtually all orexin-immunoreactive neurons are also P2X2-immunoreactive, whereas 80% of P2X2-immunoreactive neurons are also orexin-positive [330].

In addition, immunohistochemical staining also showed that P2Y1 receptors and neuronal nitric oxide synthase (nNOS) colocalize in neurons of the VMH and LH [321], two functionally antagonistic regions involved in the regulation of food intake, in which activation of VMH neurons inhibits feeding, whereas stimulation of LH neurons enhances food intake.

#### 5.10.2. P2X Activity in LHA Cells Studied by Electrophysiology

Whole-cell patch-clamp recording from LHA neurons in hypothalamic slices of transgenic mice expressing green fluorescent protein (GFP) exclusively in cells producing hypocretin/orexin showed that application of ATP depolarizes the cells and induces an increase in action potential frequency, but not frequency of spontaneous miniature postsynaptic currents [329]. In voltage clamp mode, ATP evoked a small inward somatic current that was mimicked by ATPγS, but not by αβmeATP, inhibited by suramin and PPADS, and potentiated by acid pH, suggesting P2X2 involvement [329]. In addition, suprathreshold activation of neurons of embryonic chick and postnatal mouse lateral hypothalamus in vitro triggered simultaneous release of ATP with GABA [331].

#### 5.10.3. Functional Relevance of ATP Acting at P2X in the LHA

The depolarizing response of LHA neurons to extracellular ATP could lead to an increased activity of the hypocretin arousal system [329] but, to our knowledge, the functional relevance of ATP acting at P2X has not yet been investigated for this system.

It is interesting that in the LHA there is an increase in ATP release while reducing neuronal activity during sleep [292]. Analysis of ATP content using the luciferin–luciferase-based assay in weighed and homogenized samples of rat brain tissue showed that ATP levels in the lateral hypothalamus decreased after sleep deprivation compared to a time-matched undisturbed sleeping controls [292]. A significant positive correlation was observed between ATP release and EEG delta activity during non-rapid eye movements during spontaneous sleep [292].

In addition, a direct coupling between purinergic signaling and NOS activity during adaptive feeding processes was found in rats after microinjections of P2 agonists into both VMH and LH [321]. As mentioned above (see Section 5.6.4), the authors demonstrated that food intake is dependent on P2Y1 and nitric oxide production in both regions [321].

## 6. Conclusions

The aim of this review was to summarize studies on the identification of P2X receptor genes and pathways differentially expressed in hypothalamic nuclei involved in endocrine and nonendocrine functions. Among the seven P2X isoforms described in mammalian cells (P2X1–7), the P2X2, P2X4, and P2X7 receptors were most frequently detected in the hypothalamus at both mRNA and protein levels. The P2X2 and P2X4 are mainly functional in neurons, and P2X7 in astrocytes, ependymal cells and tanycytes. Interestingly, there may be difference in the number or type of the P2X not only between hypothalamic nuclei, but also between neuronal types within individual nuclei. For example, functional P2Xs are present on vasopressin neurons, but not on oxytocin neurons in the supraoptic nucleus (SON) [226], on kisspeptin neurons, but not on GnRH neurons in the anteroventral periventricular nucleus (AVPV) [287]; on VIP neurons, but less on AVP neurons in the suprachiasmatic nucleus (SCN) [223]; and many more P2Xs are present on orexigenic AgRP/NPY neurons than on anorexigenic POMC/CART neurons and neurons containing α-MSH or DYN in the arcuate nucleus (ARC) [108]. We believe that future work will clarify these differences, as well as the involvement of P2X in specific hypothalamic functions and hypothalamic disorders.

## Abbreviations

The following abbreviations are used in this manuscript:

ADP	adenosine-5′-diphosphate
AgRP	agouti-related peptide
AMP	adenosine-5′-monophosphate
ATP	adenosine-5′-triphosphate
ATPγS	adenosine 5′-O-(2-thiotriphosphate)
2MeSATP	2-methylthio-adenosine triphosphate
AMPA	α-amino-3-hydroxy-5-methylisoxazole propionic acid
αβmeATP	α,β-methyleneadenosine 5′-triphosphate
AHN	anterior hypothalamic nucleus
ARC	arcuate nucleus
AVP	arginine vasopressin
AVPV	anteroventral periventricular nucleus
BBG	brilliant blue G
BzATP	3’-O-(4-benzoyl)benzoyladenosine-5’-triphosphate
CNS	central nervous system
DMH	dorsomedial hypothalamic nucleus
DYN	dynorphin peptide
GABA	γ-aminobutyric acid
GABA_A_	GABA receptor type A
GFP	green fluorescent protein
GnRH	gonadotropin-releasing hormone
Kiss1R	kisspeptin receptor
LHA	lateral hypothalamic area
mIPSC	miniature inhibitory postsynaptic current
mEPSC	iniature excitatory postsynaptic current
MnPO	median preoptic nucleus
nNOS	neuronal nitric oxide synthase
NMDA	N-methyl-D-aspartate
MPOA	medial preoptic area
NPP1	ecto-nucleotide pyrophosphatase/phosphodiesterase 1
NPY	neuropeptide Y
P1	purinergic receptor type 1
P2X	purinergic receptor type 2 (ionotropic)
P2Y	purinergic receptor type 2 (metabotropic)
POA	preoptic area
PPADS	pyridoxalphosphate-6-azophenyl-2′, 4′-disulfonic acid
PVN	paraventricular nucleus
SCN	suprachiasmatic nucleus
SON	supraoptic nucleus
VMH	ventromedial hypothalamic nucleus
VTM	ventral tuberomammillary nucleus
TMN	tuberomammillary nucleus
